# Trajectories of childhood eating behaviors and their association with internalizing and externalizing symptoms in adolescence

**DOI:** 10.1186/s12887-025-06001-z

**Published:** 2025-08-29

**Authors:** Rachel Dufour, Édith Breton, Sylvana M. Côté, Lise Dubois, Frank Vitaro, Michel Boivin, Richard E. Tremblay, Linda Booij

**Affiliations:** 1 Department of Psychology, Concordia University, 7141 Sherbrooke W Street, Montreal, QC H4B 1R6 Canada; 2 Eating Disorders Continuum & Douglas Research Centre, Montreal West Island Integrated Health and Social Services Centre, Douglas Mental Health University Institute, 6603 LaSalle Blvd, Verdun, QC H4H 1R3 Canada; 3 CHU Sainte-Justine Azrieli Research Center, 3175 Chem. De la Côte- Sainte-Catherine, Montreal, QC H3T 1C5 Canada; 4 Department of Fundamental Sciences, Université du Québec à Chicoutimi, 555 de l’Université Blvd, Chicoutimi, QC G7H 2B1 Canada; 5 School of Public Health, University of Montreal, 7101 du Parc Ave, Montreal, QC H3N 1X9 Canada; 6 School of Epidemiology and Public Health, University of Ottawa, 75 Laurier Ave E, Ottawa, ON K1N 6N5 Canada; 7 School of Psychoeducation, University of Montreal, 2900 Édouard- Montpetit Blvd, Montreal, QC H3T 1J4 Canada; 8 Department of Psychology, University Laval, 2325 des Bibliothèques Street, Quebec, QC G1V 0A6 Canada; 9 Department of Psychology and Pediatrics, University of Montreal, 2900 Édouard-Montpetit Blvd, Montreal, QC H3T 1J4 Canada; 10 Department of Psychiatry, McGill University, 1033 Pine Ave W, H3A 1A1, Montreal, QC Canada

**Keywords:** Childhood eating behaviors, Overeating, Picky eating, Adolescence, Trajectories, Externalizing symptoms, Internalizing symptoms

## Abstract

**Objective:**

Several studies have shown that maladaptive eating behaviors in childhood predict greater risk for eating disorders in adolescence. Whether or not maladaptive eating behaviors could represent developmental risk factors for a larger spectrum of psychopathologies is unknown. This study described longitudinal trajectories of overeating and picky eating behaviors in boys and girls from ages 2.5 to 6 years. We then examined whether these developmental trajectories in childhood are associated with internalizing and externalizing symptoms during mid-adolescence (age 15).

**Methods:**

2 014 participants were recruited at birth as part of the Quebec Longitudinal Study of Child Development. Mothers completed a measure of childhood eating behaviors at 29, 41, 44–56, 56–68 months, and 6 years old. Participants completed the Mental Health and Social Inadaptation Assessment for Adolescents at age 15. Latent class analyses and univariate regression analyses were conducted.

**Results:**

The optimal model for overeating behaviors had three trajectory groups (early-onset overeating; 14.1%, late-onset overeating; 24.3%, and never-displayed overeating; 61.6%). Three stable trajectory groups were found for picky eating behaviors (high level; 7.1%, mid-level; 37.4%, low level; 55.5%). Higher overeating behaviors in childhood were associated with greater impulsivity, hyperactivity, and anxiety in adolescence in girls but not in boys. Trajectories of picky eating were not linked with mental-health symptoms in adolescence.

**Conclusions:**

Overeating behaviors appear less stable over time than picky eating behaviors. Our findings highlight the importance of addressing psychological well-being and ADHD symptoms in children who overeat, particularly in girls, rather than focusing solely on healthy eating habits.

**Supplementary Information:**

The online version contains supplementary material available at 10.1186/s12887-025-06001-z.

## Introduction

Early childhood is a critical period for shaping long-term mental health, in part through its influence on neurodevelopment [1]. Among the many factors that contribute to this developmental trajectory, childhood eating behavior stands out as both a reflection of a child’s environment [2, 3] and a potential indicator of underlying neural processes relevant to mental well-being [4]. Children’s eating patterns vary widely, but two particularly salient behaviors have generated interest: overeating (OE) and picky eating (PE). Children who overeat tend to consume food in excess of their nutritional needs, often eating rapidly or in the absence of hunger. In contrast, children who are considered picky eaters are highly selective, resisting unfamiliar foods or accepting only a narrow range of items [4]. Other childhood eating patterns may emerge throughout development, such as various responsiveness to foods, satiety sensitivity, and slowness in eating. However, more attention has been given to OE and PE behaviors, both in research and in community interventions, partly due to their prevalence in childhood. Among children aged 2–5 years, evidence suggests that 39% present OE and 33% exhibit PE behaviors at least at some point [4].

Although OE and PE behaviors are common in childhood, key aspects of their developmental course remain poorly understood. First, studies in community samples have reported varied developmental patterns of OE and PE behaviors, including the age at which these behaviors emerge and whether they are temporary or persistent [5, 6]. Second, sex differences are still unclear; for example, one study found that males were more likely to have persistent PE [6], while other studies simply controlled for sex as a covariate in their analyses (e.g., [4, 5]). Finally, unlike some of the other early eating patterns mentioned above, OE and PE behaviors have been observed in adolescence and adulthood [7, 8]. However, potential links between early OE and PE and later outcomes, such as mental-health difficulties, remain understudied.

While OE and PE behaviors have documented associations with eating disorders [5, 9], their associations with broader dimensions of psychopathology, such as externalizing and internalizing symptoms, remain unclear. Some cross-sectional studies suggest that OE is concurrently associated with symptoms of attention deficit/hyperactivity disorder (ADHD). In contrast, PE is concurrent with symptoms of autism spectrum disorder, ADHD and obsessive-compulsive disorder [10–12]. Emotion dysregulation, which has been associated with disordered eating in adolescence [13, 14], is a potential common mechanism between certain childhood eating behaviors, like OE, and mental-health difficulties later in life. However, evidence for longitudinal associations is limited. In particular, prior studies indicate that PE behaviors before age 5 or 6 are not reliably associated with later psychopathology [15–18].

Several important gaps remain in the literature despite growing interest in the association between childhood eating behaviors and mental health. Recent studies in early childhood have focused on neurodevelopmental disorders, temperament, and self-regulation (e.g., [11],[20–22]). Thus, associations between OE and PE behaviors and the most common mental-health symptoms, anxiety and depression [19], remain understudied. Additionally, very few studies take pubertal status into account as a potential moderator, which becomes particularly relevant when examining adolescent mental health. Advanced pubertal status has been associated with mental-health problems like depression and eating disorders [23–26], with hormonal disturbances having been linked with emotion dysregulation [27–29]. Overall, understanding the overlap and specificity of potential risk factors, such as childhood eating and puberty, across different psychopathologies, represents a broader challenge in pediatric psychiatry (e.g [30]). A better understanding of the relation between early eating behaviors and common mental-health dimensions may help to support parenting interventions and psychoeducation in early childhood.

### Aims & hypotheses

The present study uses a longitudinal design to characterize the developmental course of OE and PE in childhood, and to study whether OE and PE behaviors may represent risk factors for psychopathologies in the internalized or externalized spectra in adolescence. The specific aims of this study were to (1) describe the development of OE and PE behaviors in early childhood, in a large community sample representative of the Quebec (Canada) population, from 2.5 to 6 years of age; (2) assess the association between trajectories of OE and PE behaviors in childhood and internalizing and externalizing symptoms during adolescence; and (3) examine sex patterns in OE and PE trajectories and their association with mental-health symptoms during adolescence. Considering advanced pubertal status may amplify vulnerability to mental-health difficulties, it was tested as a potential moderator of the preceding associations. We hypothesized that (1) there would be heterogeneity in the developmental course of PE and OE; (2) OE would be more strongly associated with externalizing behaviors, while PE would not be associated with later psychopathology; and (3) there would be sex differences and changes based on pubertal status in the preceding associations. As previous studies have reported differences in prevalence [31], we expected stronger associations between eating behaviors and internalizing symptoms in girls and stronger associations between eating behaviors and externalizing symptoms in boys. We also expected stronger associations in individuals with advanced stages of puberty.

## Methods

### Participants

Participants were recruited as part of the community-based cohort from the Quebec Longitudinal Study of Child Development (QLSCD). Babies were recruited randomly in 1997–1998 when they were around 5 months old, through the Quebec Master Birth Registry. Children were included in the present study if their eating behaviors were assessed at least once between the ages of 29 months and 6 years, resulting in the inclusion of *N* = 2 014 participants. All procedures were approved by the Health Research Ethics Committees of the Quebec Statistics Institute and the Sainte-Justine Hospital Research Centre Research Ethics Board (approval number 2006 − 200, 2762). Written informed consent was given by the participant’s primary caregiver at each data collection point.

### Childhood eating behaviors

Childhood eating behaviors were measured via maternal report at 29 months, 41 months, 44–56 months, 56–68 months, and 6 years old (see details on the development of the scale and its scoring at: "http://www.iamillbe.stat.gouv.qc.ca"and in [4]*)*. The eating-behaviors questionnaire was developed by members of the research team and a committee with experts (researchers and clinicians) on nutrition. It was designed for short screening to accommodate the large cohort longitudinal design of the study, and questions were based on those used in the Avon Longitudinal Study of Parents and Children [32]. The questionnaire was then piloted in an independent sample of parents with preschool-age children and found to have good reliability [4, 33, 34]. Overeating behaviors were measured with the following two items: [1] Does your child eat too fast?, and [2] Does your child eat too much?. Picky eating behaviors were measured with the following three items: [1] When she/he is at home with you for the main meal, how often does she/he eat a meal that is different from the other members of your family?; [2] In general, does she/he refuse to eat the food given to them?; and [3] In general, does she/he refuse to eat? Each item was rated as either *“never* [1]*” “rarely* [2]*” “sometimes* [3]*”* or *“often* [4]*”* by the participant’s mother or primary caregiver, except for PE item 1, which was rated as *“always* [1]*” “almost always* [2]*” “sometimes* [3]*”* or *“almost never* [4]*”*. Cronbach alphas were low (0.484 and 0.398 for OE and PE, respectively), which is unsurprising in scales with very few items [35]. The variables were re-coded as binary, as intended in the development of the scale, where a child was considered as displaying overeating if he “often” ate too much and/or at least “sometimes” ate too fast [4]. Similarly, children were considered as displaying picky eating if they “always” ate a different meal, “often” refused to eat the food prepared by their caregiver or “often” refused to eat. For both OE and PE, children who were considered to display the behaviors were given a score of 1 and children who did not display the behaviors had a score of 0, for each data collection point.

### Mental health assessment

At age 15, participants completed the Mental Health and Social Inadaptation Assessment for Adolescents. This brief measure assesses key symptoms of several psychiatric disorders occurring in the last 12 months [36]. The scales of interest for the present study were the three internalizing dimensions: Social Phobia (7 items), Generalized Anxiety (9 items), and Depression (8 items), and the three externalizing dimensions: ADHD (16 items divided into a Hyperactivity subscale (4 items), an Impulsivity subscale (6 items), and an Inattention subscale (6 items)), Conduct Disorder (4 subscales), and Oppositional Defiant Disorder (9 items). Items were answered on a three-point Likert scale, with 0 being “never true”, 1 being “sometimes true”, and 2 being “always true”. The scales have been shown to have good to excellent internal consistency (Cronbach alpha's = 0.70–0.97) [36].

### Pubertal status

Pubertal status at age 15 was measured using the five Tanner stages, also known as the Sexual Maturity Scale [37]. In this study, each participant was asked to identify pictures that align with their current stage of development. These stages describe the physical development of breasts, genitals, and pubic hair (from 1 = prepubertal to 5 = full physical maturity).

### Statistical analyses

#### Missing values

At age 29 months, 1 997 participants (49.6% girls) had data on the questionnaire about childhood eating behaviors. 1 950 participants (49.7% girls) had data at age 41 months and at age 44–56 months, 1 7 41 (50.4% girls) at 56–68 months, and 1 492 (50.8% girls) at 6 years old. Missing data were larger for the mental-health measure at age 15 (total available sample at 15: *N* = 1 443, 52.2% girls). Missing data were handled using semiparametric analyses in SAS with the maximum likelihood method of estimation. OE and PE trajectory membership was not associated with specific missing data patterns in adolescence (i.e., missing data patterns were proportional to the trajectory groups). Participants who had available data at age 15 did not differ significantly from those who did not have data on distributions of ethnicity, as well as on baseline childhood eating behaviors. More boys were missing data at age 15 (57%) than girls, while the sex distribution at baseline was equal.

#### Eating behavior trajectories

Modeling of different trajectories for childhood eating behaviors was done with the Proc Traj program of SAS (version 9.4) [38]. First, models with different numbers of trajectory groups are tested to identify the best number to fit the data (e.g., 1, 2, 3, and/or 4 groups). Second, models with different trajectory shapes (e.g., constant, linear, quadratic, and cubic) are tested to identify the best shape for each trajectory. Choices for best models are guided by the Bayesian Information Criterion (BIC), graphic inspection and mean posterior probabilities for group membership. Posterior probabilities represent a participant’s probability of being assigned to the trajectory group. For this study, trajectories for OE and PE in childhood were modeled separately. Correlations and a chi-square test were conducted to assess the concordance between OE and PE trajectories.

#### Association between OE and PE trajectories and mental health

Due to the lack of normality of certain mental-health variables, data transformations were done before analysis. Based on indices of skewness, kurtosis, and visual inspection, a square root transformation was found to be adequate for social phobia, hyperactivity, and conduct problems. A log transformation offered the best transformation for opposition. Original forms were retained for impulsivity, inattention, anxiety, and depressive symptoms. Additional File 1 displays the descriptive statistics of the mental-health variables. 

Next, univariate linear regression analyses with group membership, sex, and their interaction term as predictors of internalizing (i.e., depression, anxiety, social phobia) or externalizing (i.e., ADHD, conduct disorder, oppositional defiant disorder) symptoms were conducted in SPSS (version 27). To allow for variability in ADHD symptoms to be considered in the analyses, the three ADHD subscales were analyzed as separate outcomes. Trajectory group membership was coded as follows for these analyses: 1 = “never displayed OE” or “never displayed PE”, 2 = “early-onset OE” or “mid-level PE”, and 3 = “late-onset OE” or “high-level PE” (see Results for descriptions of the trajectory modelling results). Then, univariate regression analyses stratified by sex were performed with OE or PE trajectory group membership as predictors, and the relevant scales of the Mental Health and Social Inadaptation Assessment for Adolescents as outcomes. Dummy coding was used for sex (i.e., female = 0, male = 1). Using the PROCESS macro in SPSS [39], supplementary analyses controlling for BMI at age 15 and to test the potential moderating role of pubertal status on the regression analyses were also conducted. A Bonferroni correction was applied to correct for multiple comparisons (corrected alpha set at 0.006).

## Results

### Demographics

At each time point, between 49.6% and 50.8% of the sample was composed of girls. Nationality was distributed in the following way: 72.9% (*n* = 1 415) Canadian, 32.7% (*n* = 635) French, 7.1% (*n* = 138) British, 8.9% (*n* = 167) European, 2.8% (*n* = 54) native, 2% (*n* = 39) African or Haitian, and 12.9% (*n* = 250) other.

### Trajectories of childhood OE

**Fig. 1:**
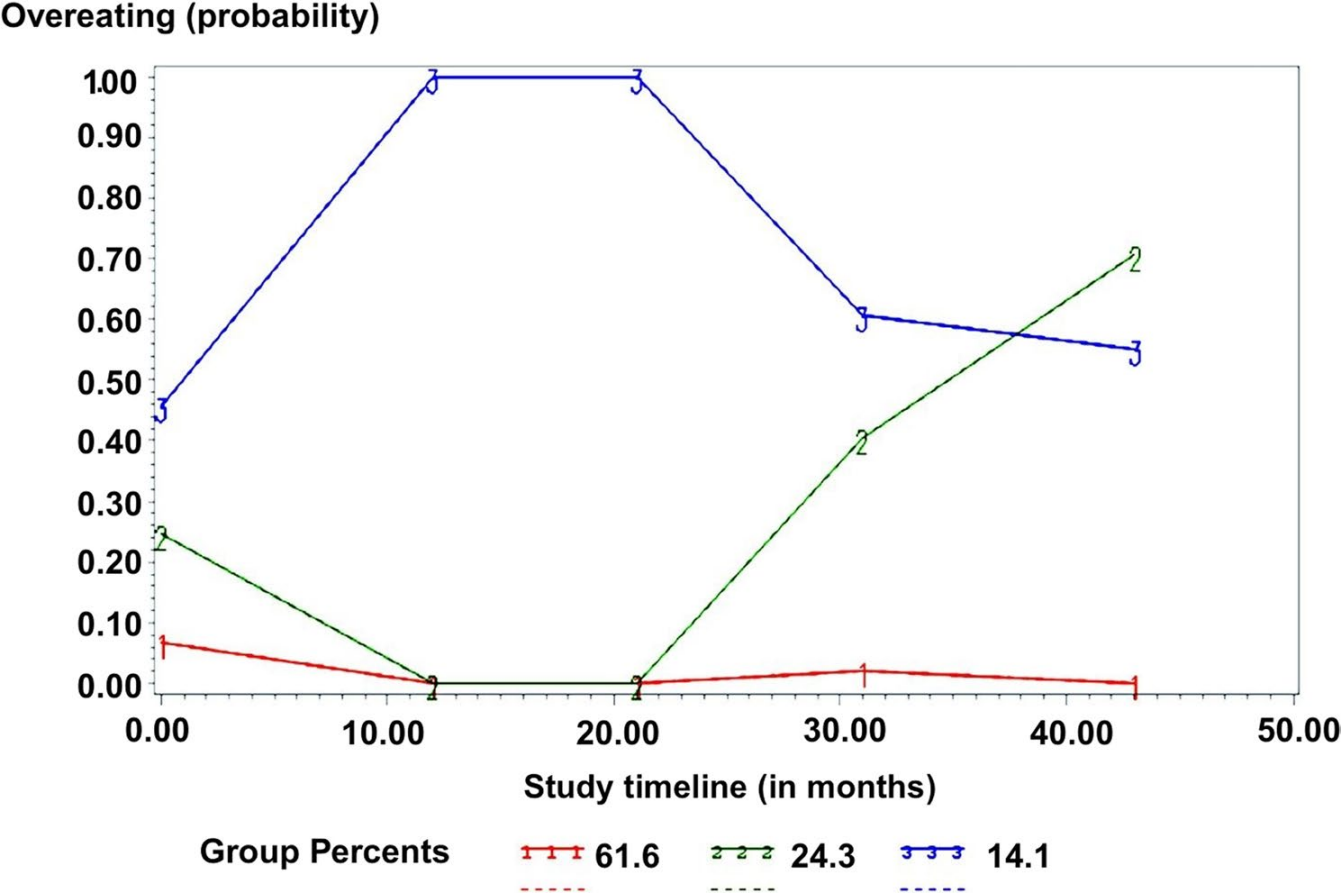
Selected trajectories of OE behaviors as a function of time. Overeating trajectories from 29 months to 6 years of age. The dashed lines represent estimated values, whereas the solid lines represent observed values. X-axis represents the study timeline in months whereas Y-axis represents the probability of presenting OE behaviors

The process of identifying the best model fit for trajectories of OE and PE behaviors is shown in Additional File 2. The optimal model for OE behaviors had three trajectory groups, each of them best defined by cubic equations. There was an “early-onset OE” group (14.1% of the cohort), a “late-onset OE” group (24.3% of the cohort), and a “never displayed OE” group (61.6% of the cohort). The “early-onset OE” group started high with a large increase between 29 and 41 months, a decline between 44 and 56 months and 56–68 months, and remained stable towards 6 years old (*p* <0.05). The “late-onset OE” group started relatively low but had a sharp increase in OE starting at 44–56 months (*p* >0.05). The “never displayed OE” group stayed low with slight variations over time (*p* <0.01). Figure 1 shows the selected model of three trajectories for OE behavior across early childhood in the cohort. Mean posterior probabilities were 0.89 (*SD* = 0.09) for the “early-onset OE” group, 0.91 (*SD* = 0.15) for the “late-onset OE” group, and 0.98 (*SD* = 0.11) for the “never displayed OE” group, which represents good fit, according to the guidelines by Nagin et al. [40].

### Trajectories of childhood PE

**Fig. 2:**
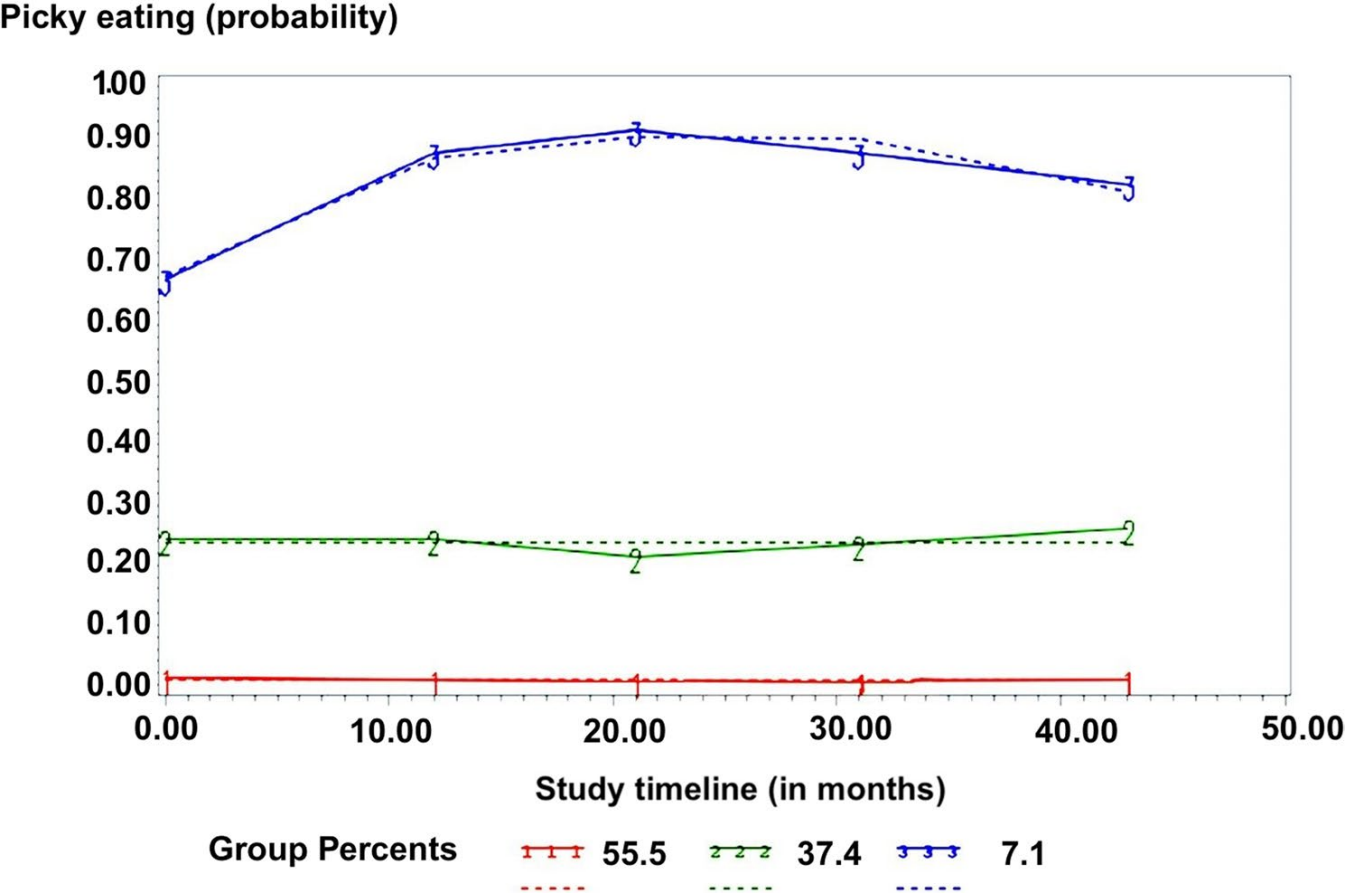
Selected trajectories of PE behaviors as a function of time. Picky-eating trajectories from 29 months to 6 years of age. The dashed lines represent estimated values, whereas the solid lines represent observed values. X-axis represents the study timeline in months whereas Y-axis represents the probability of presenting PE behaviors

The optimal model for childhood PE behaviors had three trajectories (*p* <0.01), with two being defined by a constant and one being defined by a quadratic equation. There was a “high-level” trajectory group (7.1% of the cohort), a “mid-level” trajectory group (37.4% of the cohort), and a “low-level” trajectory group (55.5% of the cohort). The high-level trajectory group started high at 29 months and remained high, with a slight decrease over time. The mid-level trajectory had a constant level of PE behaviors over time. The low-level trajectory group remained low throughout early childhood. Figure 2 shows the selected model with three trajectory groups of PE behaviors in the cohort. Mean posterior probabilities were 0.83 (*SD* = 0.04) for the first group, 0.87 (*SD* = 0.10) for the second group, and 0.99 (*SD* = 0.13) for the third group, demonstrating good fit, following the Nagin et al. (1999) guidelines [40].

The correlation between OE and PE trajectories (Spearman’s rho = 0.08) was low. 42.3% of participants did not display any OE or PE behaviors, 5% displayed high levels of PE with no OE, 12.3% displayed early-onset OE with no PE, and 10.1% displayed late-onset OE with no PE. A chi-square test revealed a statistically significant difference between OE and PE membership proportions (χ² (1) = 10.9, *p* =0.001), although the effect size was very small (V = 0.09).

### Longitudinal associations between OE and PE and mental health

Results of the regression analysis can be found in Table 1. Trajectories of OE did not account for a significant portion of variance explained in any mental-health symptoms. In the whole-sample analyses, sex accounted for a statistically significant portion of the variance explained in social phobia, hyperactivity, generalized anxiety, and depression in adolescence. There was a statistically significant interaction between sex and OE trajectory for hyperactivity and for impulsivity before the multiple-comparison correction was applied, with late-onset OE behaviors being associated with higher impulsivity and hyperactivity in girls but not in boys.

**Table 1:** Univariate regression analyses with trajectory group and sex interactions

	Model	Group			Sex			Group * Sex		
	*R*^*2*^	*B* [CI]	β	*p*	*B* [CI]	β	*p*	*B* [CI]	β	*p*
***Overeating behaviors***										
Social phobia	0.063**	0.04 [−0.04 – 0.12]	0.03	0.378	**0.36** [0.17–0.55]	**0.213**	**< 0.001**	0.04 [−0.07 – 0.16]	0.04	0.480
Impulsivity	0.010*	−0.01 [−0.20 – 0.19]	− 0.002	0.957	−0.38 [−0.84 – 0.08]	− 0.096	0.109	**0.37** [0.09–0.64]	**0.161**	**0.010**
Hyperactivity	0.009**	−0.02 [−0.10 – 0.06]	− 0.018	0.628	**−0.24** [−0.44– − 0.05]	**− 0.146**	**0.015**	**0.18** [0.06–0.29]	**0.183**	**0.004**
Inattention	0.010*	−0.01 [−0.20 – 0.19]	− 0.003	0.937	0.06 [−0.4 – 0.52]	0.015	0.795	0.20 [−0.08 – 0.49]	0.089	0.154
Generalized anxiety	0.137**	0.03 [−0.17 – 0.23]	0.009	0.782	**1.20** [0.72–1.68]	**0.276**	**< 0.001**	0.28 [−0.01 – 0.56]	0.109	0.062
Conduct	0.003	−0.01 [−0.06 – 0.05]	− 0.005	0.896	−0.08 [−0.21 – 0.05]	− 0.074	0.214	0.06 [−0.02 – 0.14]	0.099	0.117
Depression	0.136**	0.04 [−0.16 – 0.25]	0.014	0.680	**1.29** [0.8–1.79]	**0.288**	**< 0.001**	0.24 [−0.06 – 0.54]	0.094	0.110
Opposition	0.002	0.02 [−0.02 – 0.06]	0.035	0.342	−0.01 [−0.12 – 0.09]	− 0.016	0.793	0.01 [−0.05 – 0.07]	0.025	0.695
***Picky eating behaviors***										
Social phobia	0.060**	−0.03 [−0.13 – 0.08]	− 0.018	0.631	**0.34** [0.12–0.55]	**0.201**	**0.002**	0.05 [−0.09 – 0.19]	0.071	0.461
Impulsivity	0.002	−0.04 [−0.29 – 0.20]	− 0.013	0.736	0.24 [−0.28 – 0.75]	0.060	0.370	−0.07 [−0.40 – 0.27]	− 0.029	0.691
Hyperactivity	0.002	−0.05 [−0.15 – 0.05]	− 0.037	0.340	0.03 [−0.19 – 0.25]	0.019	0.781	−0.02 [−0.16– 0.13]	− 0.016	0.826
Inattention	0.008*	−0.04 [−0.29 – 0.20]	− 0.013	0.734	0.29 [−0.23 – 0.81]	0.072	0.280	0.05 [−0.29 – 0.38]	0.019	0.793
Generalized anxiety	0.133**	−0.06 [−0.31 – 0.20]	− 0.015	0.671	**1.63** [1.09–2.16]	**0.374**	**< 0.001**	−0.03 [−0.38– 0.32]	− 0.012	0.855
Conduct	0.000	−0.02 [−0.09 – 0.05]	− 0.025	0.516	−0.02 [−0.16 – 0.12]	− 0.018	0.787	0.02 [−0.07 – 0.11]	0.029	0.690
Depression	0.132**	0.02 [−0.24 – 0.29]	0.006	0.862	**1.66** [1.1–2.21]	**0.368**	**< 0.001**	−0.02 [−0.38– 0.34]	− 0.007	0.923
Opposition	0.000	−0.02 [−0.07 – 0.04]	− 0.025	0.515	−0.02 [−0.14– 0.09]	− 0.027	0.692	0.02 [−0.06 – 0.09]	0.033	0.655

Statistically significant regression coefficients have been bolded. CI represent 95% confidence intervals for the unstandardized coefficients. B indicates the change in the dependent variable for a one-unit increase in the predictor. β indicates the change in the dependent variable, in standard deviations, for one standard deviation increase in the predictor. Each internalizing or externalizing symptom dimension was regressed on group membership of either overeating or picky eating, sex, and their interaction term

* = uncorrected *p* <0.01

** = Bonferroni corrected *p* <0.006

PE did not account for a significant portion of variance explained in any mental-health symptoms. Sex significantly accounted for variance explained in social phobia, generalized anxiety, and depression. No interactions were found between PE behaviors and sex.

### Sex-stratified associations

Results of sex-stratified regression analyses can be found in Table 2. In girls, OE behaviors (top section of Table 2) were positively associated with generalized anxiety symptoms, in addition to impulsivity and hyperactivity. The preceding associations were not present in boys. Trajectories of PE behaviors (bottom section of Table 2) were not statistically significant predictors of any mental-health outcomes in either girls or boys.

**Table 2:** Univariate regression analyses of childhood eating trajectories and adolescent mental-health symptoms

	Girls			Boys		
	*R*^*2*^	*B* [CI]	β	*R*^*2*^	*B* [CI]	β
***Overeating behaviors***						
Social phobia	0.004	0.08 [−0.01 – 0.16]	0.07	0.001	0.04 [−0.05 – 0.12]	0.03
Impulsivity	0.016**	**0.35** [0.16–0.54]	**0.13**	− 0.001	−0.01 [−0.21 – 0.20]	− 0.002
Hyperactivity	0.014**	**0.15** [0.07–0.24]	**0.13**	1.8 × 10-5	−0.02 [−0.1 – 0.06]	− 0.02
Inattention	0.005	0.19 [−0.01 – 0.40]	0.067	9 × 10-6	−0.01 [−0.2 – 0.18]	− 0.003
Generalized anxiety	0.010**	**0.30** [0.09–0.51]	**0.10**	1.2 × 10-4	0.03 [−0.17 – 0.23]	0.11
Conduct	0.005*	**0.06** [0.01–0.11]	**0.074**	3 × 10-6	−0.01 [−0.06 – 0.05]	− 0.005
Depression	0.008*	**0.28** [0.05–0.51]	**0.089**	2.9 × 10-4	0.04 [−0.15 – 0.24]	0.017
Opposition	0.003	0.03 [−0.01 – 0.08]	0.053	0.001	0.02 [−0.02 – 0.06]	0.036
***Picky eating behaviors***						
Social phobia	0.001	0.03 [−0.07 – 0.12]	0.021	2.4 × 10-4	−0.03 [−0.13 – 0.07]	− 0.018
Impulsivity	0.001	−0.11 [−0.33 – 0.11]	− 0.036	1.5 × 10-4	−0.04 [−0.29 – 0.21]	− 0.012
Hyperactivity	0.002	−0.07 [−0.16 – 0.03]	− 0.05	0.001	−0.05 [−0.15 – 0.05]	− 0.036
Inattention	5.0 × 10-7	0.01 [−0.24 – 0.24]	0.001	1.8 × 10-4	−0.04 [−0.28 – 0.20]	− 0.013
Generalized anxiety	0.001	−0.09 [−0.33 – 0.16]	− 0.026	2.8 × 10-4	−0.05 [−0.30 – 0.19]	− 0.17
Conduct	3.8 × 10-4	−0.01 [−0.07 – 0.06]	− 0.004	0.001	−0.02 [−0.09 – 0.04]	− 0.025
Depression	2 × 10-6	0.01 [−0.26 – 0.27]	0.002	5.1 × 10-5	0.02 [−0.22 – 0.26]	0.007
Opposition	2 × 10-5	−0.01 [−0.05 – 0.05]	− 0.002	0.001	−0.02 [−0.07 – 0.04]	− 0.025

CI represent 95% confidence intervals for the unstandardized coefficients. B indicates the change in the dependent variable for a one-unit increase in the predictor. β indicates the change in the dependent variable, in standard deviations, for one standard deviation increase in the predictor. Within either the girls or boys sample, each internalizing or externalizing symptom dimension was regressed on group membership of either overeating or picky eating

* = uncorrected *p* <0.05

** = Bonferroni corrected *p* <0.006

Pubertal status as a moderator was tested for both sexes (see Additional File 3). After applying the Bonferroni correction, pubertal status was not a statistically significant moderator of any of the associations between childhood eating behaviors and mental-health symptoms in girls or boys.

### Sensitivity analyses

Separate regression analyses controlling for posterior probabilities and for BMI at age 6 were conducted to verify the potential impacts of both variables on the results. Posterior probabilities were found to have little impact (i.e., changes smaller than 0.01 and no change in the statistical significance) on the regression results (i.e., on the *R*^*2*^, the *p* values, and the coefficients), for both the full sample and the sex-stratified results and did not predict any of the outcomes. Similarly, adding BMI to the model had no impact on the associations between trajectory membership and mental health in adolescence. BMI at age 6 was also not a statistically significant predictor of any of the mental-health measures at age 15.

## Discussion

This study described longitudinal developmental trajectories of OE and PE behaviors in children from 2.5 years to 6 years of age and examined associations with mental-health symptoms in mid-adolescence. Findings showed different trajectories for OE and PE behaviors in early childhood, with more stable trajectories for PE than for OE behaviors. OE behaviors, but not PE behaviors, were associated with impulsivity, hyperactivity, and symptoms of anxiety in girls but not in boys.

The developmental trajectories identified for OE and PE behaviors largely align with prior research, while also offering possible new insights into the patterns and prevalence of OE and PE behaviors over time. Notably, two distinct OE trajectories emerged (an early onset and a later onset), mirroring earlier studies that similarly distinguished between early and late manifestations of these behaviors [41]. As for PE behaviors, the prevalence of our trajectories is consistent with the prevalence of low-level and persistent PE from Cano and colleagues (2015) [6]. However, our results differ from previous work by identifying a greater proportion of children in trajectories that may represent elevated risk for OE [5, 41]. We also documented more stable PE trajectories than have previously been reported, including among children with higher levels of PE behavior [6, 15].

Trajectories of OE behaviors were associated with two dimensions of ADHD: impulsivity and hyperactivity. Stratified analyses showed that OE behaviors in girls were related to higher levels of adolescent impulsivity and hyperactivity, despite boys presenting with higher levels of both symptom dimensions. Among possible contributing factors to these associations that should be further explored, two potential explanatory mechanisms seem particularly relevant: inhibitory control and emotion regulation. Some research suggests that poor inhibitory control is associated with OE behaviors and loss of control over eating (e.g., [42, 43]). Similarly, children with difficulties regulating their emotions may be at a higher risk of engaging in OE behaviors [44]. Dysregulation in emotions and inhibition, which have been documented in ADHD (e.g., [45, 46]), could thus explain the presentation of more OE behaviors. Our results, along with previous literature, potentially suggest that OE behaviors could serve as early behavioral markers for later ADHD symptoms.

In addition to associations with externalizing symptoms, OE behaviors in childhood were also associated with symptoms of anxiety in girls. Anxiety symptoms tend to be more prevalent in girls and women than in boys and men [47, 48], and have been previously associated with emotional eating [49]. OE behaviors may represent an early sign of these types of emotional difficulties in girls. However, as early mental-health measures in childhood were not available, it is unclear whether adolescent mental-health symptoms reflect new difficulties or a continuation of earlier symptoms. Future longitudinal studies including early mental health measures are needed to further clarify these associations.

Several developmental and sociocultural factors may help explain the observed sex differences (i.e., lack of associations in boys). Although advanced pubertal status was considered a potential explanatory mechanism, our results did not yield any significant moderating effects. Sociocultural influences, such as how children are socialized around food and parental feeding practices, could partly explain observed sex differences. Parents tend to be more restrictive in their feeding practices with girls, while boys are often encouraged to eat more, which could translate into an increased risk for disordered eating, including OE behaviors, and mental-health difficulties in girls [50, 51]. More research is needed to further investigate the developmental and environmental mechanisms driving such sex differences.

In contrast to OE behaviors, PE trajectories in early childhood were not associated with mental-health symptom dimensions in the present study. These results confirm prior research findings [15–18] and expand evidence to symptoms of anxiety and depression. Future research should consider incorporating assessments of other types of mental-health conditions to clarify the scope and specificity of PE’s associations with later outcomes. For example, previous studies have suggested possible associations with neurodevelopmental disorders such as autism spectrum disorder, a condition with a high prevalence of food sensitivity and restriction [20, 52]. It is therefore possible that PE behaviors are primarily associated with restrictive eating and neurodevelopmental conditions.

The present study has several strengths. To our knowledge, this study is a first to examine early childhood eating behaviors as potential risk factors for depressive and anxiety symptoms in a large community sample of individuals. The longitudinal cohort design over a significant period allowed for the analysis of these associations. Our study design permitted us to measure a wide range of mental-health symptoms, both within the externalizing and internalizing dimensions. The nature of our sample allowed us to conduct sex-specific analyses, which further improves the generalizability of our findings. Additionally, the use of semi-parametric trajectory analyses with embedded maximum likelihood estimation minimized the impact of missing data on the results.

Some limitations should be considered when interpreting our results. The scale used to measure childhood eating behaviors was reported by the mother; thus, it could be subject to bias, as the parents significantly influence children’s food intake in early childhood. For OE specifically, the reliance on only two behavioral indicators also limits the definition of this category of childhood eating behaviors. The small number of items to assess childhood eating behaviors and the dichotomization of responses to calculate the trajectories (into those who displayed PE or not, and OE or not), as the scale was originally designed for and used in previous studies [4, 9, 34, 53], could have removed relevant variability between individuals. Still, this practical, short scale allowed for multiple early assessments in childhood, its use has led to several contributions to the field of child development [4, 9, 34, 53], and its predictive validity has been supported by a recent study, showing that childhood PE and OE behaviors were linked with disordered eating in young adulthood [33]. Although our statistical methods accounted for missing data, there were large differences between the amount of missing data in childhood and in adolescence. Additionally, considering our community-based sample, it is unclear if the associations we found between OE behaviors and mental health in girls also generalize to clinical populations. Finally, with relatively small effect sizes, our study warrants replication. Our results in a community-based sample provide a basis for future work in preventive health, and if replicated, could eventually be used to help promote health and well-being in youth.

## Conclusions

Our study showed distinct developmental trajectories for OE and PE behaviors, indicating that childhood OE behaviors may be less stable over time than PE. Both eating behaviors appear to be common during early childhood. Childhood OE behaviors in girls was associated with greater impulsivity, hyperactivity, and anxiety in adolescence. Whereas OE in children may be generalized as a risk for obesity, our findings highlight that these children, girls in particular, may benefit from broader interventions that promote psychological well-being and target potential ADHD symptoms.

## Supplementary Information

Supplementary Material 1.

Supplementary Material 2.

Supplementary Material 3.

## Data Availability

The data underlying this article cannot be shared publicly due to the privacy of individuals that participated in the study. The data will be shared on reasonable request to the corresponding author (LB).

## Abbreviations

ADHD: Attention Deficit and Hyperactivity Disorder
BIC: Bayesian Information Criterion
BMI: Body mass index
DSM-V: Diagnostic and Statistical Manual of Mental Disorders, Fifth Edition
OE: Overeating
PE: Picky eating
SPSS: Statistical Package for the Social Sciences
