# Supplementary material for: Trajectories of childhood eating behaviors and their association with internalizing and externalizing symptoms in adolescence
Source: BMC Pediatr. 2025 Aug 29;25:663. doi: 10.1186/s12887-025-06001-z (PMC12395660; doi:10.1186/s12887-025-06001-z)
Supplement: Supplementary file 1 — Supplementary Material 1. [file 12887_2025_6001_MOESM1_ESM.docx]

**Supplementary Table 1**

*Descriptive statistics of adolescent mental-health measures after data transformations.*

|  | **N (% missing)** | **Transformation used** | **Mean** | ***SD*** | **Range** | **Skewness** | **Kurtosis** |
| --- | --- | --- | --- | --- | --- | --- | --- |
| ***Internalizing behaviors*** | | | | | | | |
| **Social phobia** | 1443 (28) | Square root | 1.30 | .84 | 0–3.16 | -.189 | -.874 |
| **Generalized anxiety** | 1443 (28) | - | 4.10 | 2.17 | 0–10 | .270 | -.617 |
| **Depression** | 1442 (28) | - | 3.49 | 2.25 | 0–10 | .555 | -.129 |
| ***Externalizing behaviors*** | | | | | | | |
| **Impulsivity** | 1440 (29) | - | 2.78 | 1.95 | 0–10 | .513 | .079 |
| **Hyperactivity** | 1441 (28) | Square root | 1.39 | .83 | 0–3.16 | -.420 | -.696 |
| **Inattention** | 1442 (28) | - | 3.31 | 1.97 | 0–10 | .330 | -.304 |
| **Conduct** | 1441 (28) | Square root | .596 | .54 | 0–3.11 | .599 | .143 |
| **Opposition** | 1441 (28) | Log | 1.12 | .43 | 0–2.29 | -.352 | -.003 |
