# Supplementary material for: Trajectories of childhood eating behaviors and their association with internalizing and externalizing symptoms in adolescence
Source: BMC Pediatr. 2025 Aug 29;25:663. doi: 10.1186/s12887-025-06001-z (PMC12395660; doi:10.1186/s12887-025-06001-z)

**OVEREATING TRAJECTORIES**

**Step 1:** Identify the ideal number of groups of trajectories (best in green).

| **Model** | **# of groups** | **Order (0=constant, 1=linear, 2=quadratic, 3=cubic)** | **BIC** |
| --- | --- | --- | --- |
| 1 | 3 | 3 3 3 | -3090.90 |
| 2 | 4 | 3 3 3 3 | -3113.13 |
| 3 | 2 | 3 3 | -3178.33 |
| 4 | 1 | 3 | -4197.06 |

**Step 2:** Identify the best shape for the trajectories (best in green).

| **Model** | **# of groups** | **Order (0=constant, 1=linear, 2=quadratic, 3=cubic)** | **BIC** |
| --- | --- | --- | --- |
| 1 | 3 | 3 3 3 | -3090.90 |
| 4 | 3 | 3 2 3 | -3089.27 |
| 5 | 3 | 3 1 3 | -3295.78 |
| 6 | 3 | 3 2 2 | -3332.18 |
| 7 | 3 | 2 2 2 | -3327.62 |
| 8 | 3 | 3 3 2 | -3199.10 |
| 9 | 3 | 2 2 3 | -3231.30 |
| 10 | 3 | 1 2 3 | -3291.01 |
| 11 | 3 | 0 2 3 | -3264.32 |
| 12 | 3 | 0 3 3 | -3092.40 |
| 13 | 3 | 1 3 3 | -3096.96 |
| 14 | 3 | 2 3 3 | -3086.82 |

**Estimates for final solution:**

**
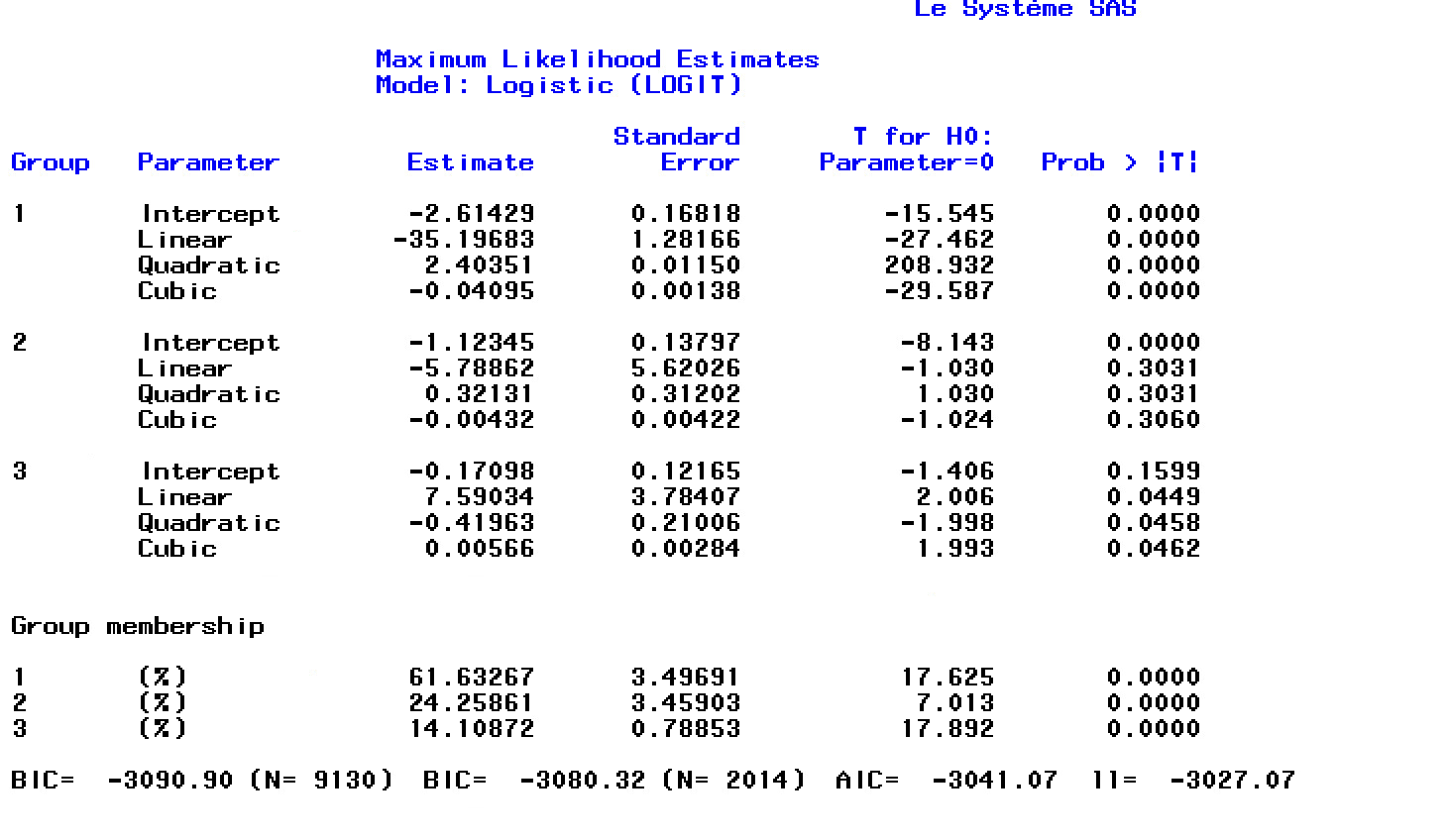
**

**PICKY-EATING TRAJECTORIES**

**Step 1:** Identify the ideal number of groups of trajectories.

| **Model** | **# of groups** | **Order (0=constant, 1=linear, 2=quadratic, 3=cubic)** | **BIC** |
| --- | --- | --- | --- |
| 1 | 3 | 3 3 3 | -3388.79 |
| 2 | 4 | 3 3 3 3 | -3401.79 |
| 3 | 2 | 3 3 | -3473.55 |
| 4 | 1 | 3 | -3937.57 |

**Step 2:** Identify the best shape for the trajectories.

| **Model** | **# of groups** | **Order (0=constant, 1=linear, 2=quadratic, 3=cubic)** | **BIC** |
| --- | --- | --- | --- |
| 1 | 3 | 3 3 3 | -3388.79 |
| 2 | 3 | 2 3 3 | -3384.50 |
| 3 | 3 | 1 3 3 | -3379.80 |
| 4 | 3 | 0 3 3 | -3379.07 |
| 5 | 3 | 1 2 3 | -3376.25 |
| 6 | 3 | 0 2 3 | -3377.03 |
| 7 | 3 | 0 1 3 | -3371.49 |
| 8 | 3 | 1 1 3 | -3371.57 |
| 9 | 3 | 1 0 3 | -3371.49 |
| 10 | 3 | 0 0 3 | -3370.10 |
| 11 | 3 | 0 0 2 | -3366.65 |
| 12 | 3 | 0 0 1 | -3368.69 |
| 13 | 3 | 0 1 2 | -3371.09 |
| 14 | 3 | 1 0 2 | -3368.03 |

**Estimates for final solution:**


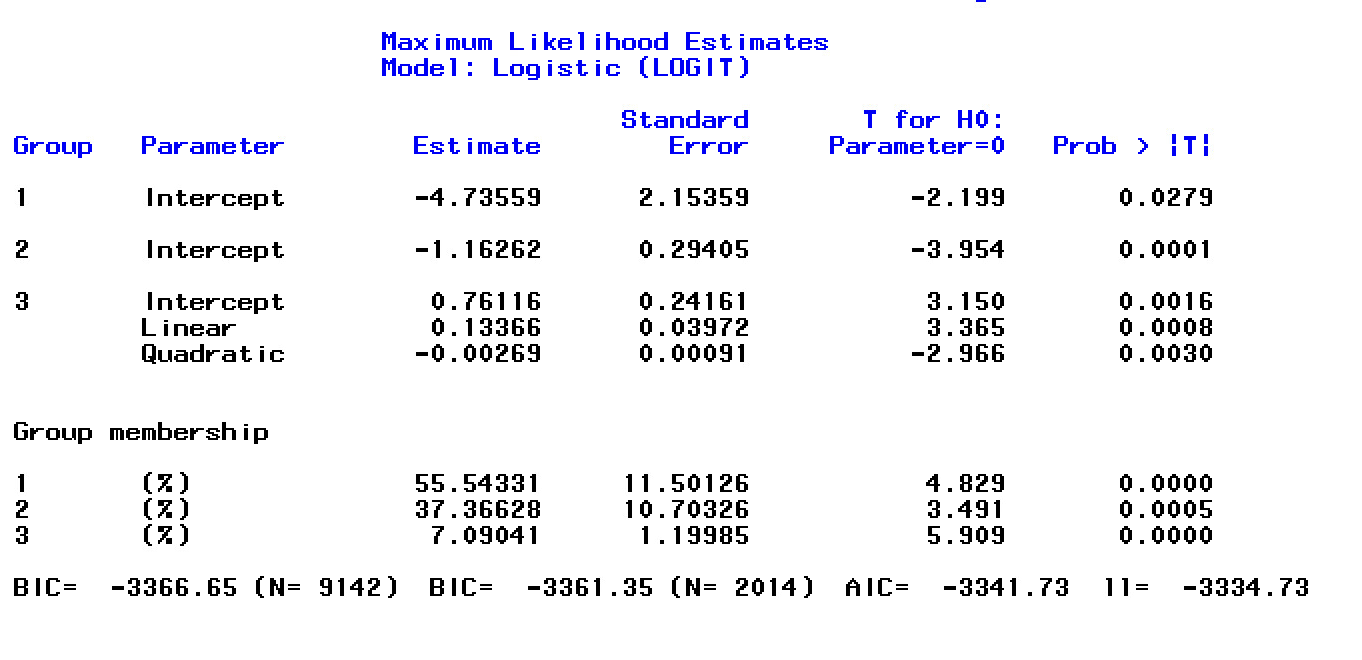

Supplement: Supplementary file 2 — Supplementary Material 2. [file 12887_2025_6001_MOESM2_ESM.docx]
