# Supplementary material for: Trajectories of childhood eating behaviors and their association with internalizing and externalizing symptoms in adolescence
Source: BMC Pediatr. 2025 Aug 29;25:663. doi: 10.1186/s12887-025-06001-z (PMC12395660; doi:10.1186/s12887-025-06001-z)
Supplement: Supplementary file 3 — Supplementary Material 3. [file 12887_2025_6001_MOESM3_ESM.docx]

**Supplementary Table 2**

*Moderation analysis results with puberty and interactions with childhood behaviors*

|  | **Girls** | | | **Boys** | | |
| --- | --- | --- | --- | --- | --- | --- |
|  | Pub. Coeff. | Interaction | *R^2^* | Pub. Coeff. | Interaction | *R^2^* |
| ***Overeating behaviors*** | | | | | | |
| **Social phobia** | -0.08 | 0.04 | .007 | -0.04 | -0.02 | .007 |
| **Impulsivity** | 0.10 | 0.11 | **.038**** | 0.10 | -0.03 | .001 |
| **Hyperactivity** | -0.01 | 0.03 | **.025**** | 0.17 | -0.09 | .006 |
| **Inattention** | 0.13 | 0.05 | **.015*** | 0.40 | -0.21 | .006 |
| **Generalized anxiety** | 0.14 | 0.12 | **.027**** | 0.20 | -0.10 | .003 |
| **Conduct** | 0.07 | -0.01 | **.016*** | 0.09 | -0.01 | **.014*** |
| **Depression** | 0.19 | 0.08 | **.023**** | 0.37 | -0.15 | .008 |
| **Opposition** | 0.02 | 0.01 | **.013*** | 0.08 | -0.04 | .007 |
| ***Picky Eating behaviors*** | | | | | | |
| **Social phobia** | 0.05 | -0.05 | .003 | **-0.27*** | **0.15*** | .012 |
| **Impulsivity** | **0.59*** | -0.24 | **.019*** | -0.45 | **-0.37*** | .009 |
| **Hyperactivity** | 0.01 | 0.03 | .008 | -0.01 | 0.02 | .002 |
| **Inattention** | 0.47 | -0.20 | .010 | -0.44 | **0.37*** | .010 |
| **Generalized anxiety** | 0.23 | 0.07 | **.018*** | -0.15 | 0.14 | .002 |
| **Conduct** | **0.20*** | **-0.11*** | **.015*** | 0.01 | 0.05 | **.018*** |
| **Depression** | 0.38 | -0.05 | **.014*** | -0.16 | 0.21 | .007 |
| **Opposition** | **0.13*** | -0.07 | .011 | -0.02 | 0.03 | .003 |

*Note.* Interaction stands for “interaction terms”. Pub stands for “puberty”. * = uncorrected

*p* < .05. ** = Bonferroni corrected *p* < .006.
